# Low-Cost Turbidity Sensor to Determine Eutrophication in Water Bodies

**DOI:** 10.3390/s23083913

**Published:** 2023-04-12

**Authors:** Javier Rocher, Jose M. Jimenez, Jesus Tomas, Jaime Lloret

**Affiliations:** Instituto de Investigación para la Gestión Integrada de Zonas Costeras, Universitat Politècnica de València, C/Paraninf, 1 Grao de Gandia, 46730 Valencia, Spain; jarocmo@doctor.upv.es (J.R.);

**Keywords:** solids, arduino, RGB LED, infrared LED, environmental pollution

## Abstract

Eutrophication is the excessive growth of algae in water bodies that causes biodiversity loss, reducing water quality and attractiveness to people. This is an important problem in water bodies. In this paper, we propose a low-cost sensor to monitor eutrophication in concentrations between 0 to 200 mg/L and in different mixtures of sediment and algae (0, 20, 40, 60, 80, and 100% algae, the rest are sediment). We use two light sources (infrared and RGB LED) and two photoreceptors at 90° and 180° of the light sources. The system has a microcontroller (M5stacks) that powers the light sources and obtains the signal received by the photoreceptors. In addition, the microcontroller is responsible for sending information and generating alerts. Our results show that the use of infrared light at 90° can determine the turbidity with an error of 7.45% in NTU readings higher than 2.73 NTUs, and the use of infrared light at 180° can measure the solid concentration with an error of 11.40%. According to the determination of the % of algae, the use of a neural network has a precision of 89.3% in the classification, and the determination of the mg/L of algae in water has an error of 17.95%.

## 1. Introduction

The environment is altered due to the impact of human activity such as agriculture, mining, etc. One of the areas that causes the most significant concern is the pollution of rivers, lakes, and seas. Water is one of the primary human survival resources; therefore, it is vitally important to have mechanisms and resources that allow us to monitor water quality continuously. By controlling turbidity, we can know the degree of water contamination. Turbidity is a measure of water quality, which describes the degree of ‘cloudiness’ of suspended particles [1].

A reduction in pollution is essential for our planet, both from an environmental and health point of view, so we need to take measurements quickly and with high reliability [2]. Most of the actions to detect turbidity in the water are carried out by taking samples later directed to laboratories. In these laboratories, there is the presence of trained personnel and corresponding instrumentation, and they provide highly accurate and precise means [3] but do not allow continuous sampling of the resources.

Although commercial solutions have appeared in the past that can provide on-site measurement [4], these devices were costly. There are authors such as [5] who, for a few years, have declared that detection carried out with low-cost sensors is viable today.

According to the ISO 7027-1 standard (Water quality—Determination of turbidity—Part 1: Quantitative methods) [6], there are two quantitative methods for determining water turbidity: nephelometry and turbidimetry.

Nephelometry is a procedure for measuring diffuse radiation in water. For this, a beam of light is projected, and the light reflected by suspended particles is measured. For this, the optical sensor is located at an angle close to 90 degrees. This technique allows for shallow turbidity values to be calculated, so it is frequently used in potable water. The measurements obtained using this method are expressed in nephelometric turbidity units (NTUs). They usually take values in the range of 0.05 NTUs–400 NTUs.

Turbidimetry is a method for measuring the attenuation of a beam of light as it passes through the fluid. In this case, the emitter and receiver are located at an angle of 180 degrees. This technique is effective in waters with high turbidities, such as wastewater. The measurements obtained using this method are expressed in formazin attenuation units (FAUs). They typically take values between 40 FAUs and 4000 FAUs.

In this paper, a low-cost turbidimeter is designed and developed. Our turbidity sensor will be able to measure the turbidity of solid substances dissolved in water. Its application can be carried out in any type of fresh and salty water, in rivers, reservoirs, irrigation rafts, etc. The main objective is detecting an excess of solids that can affect the environment, for example, the appearance of waste discharges. The sensor will use an RPG infrared LED that will be incident on a photodiode at angles of 90° and 180°.

The rest of the paper is organized as follows. Section 2 presents the related work on other works presented by different authors. Section 3 describes the Materials and Methods employed in the implementation of the work. Results are presented and discussed in Section 4. Finally, the conclusion and future work are presented in Section 5.

## 2. Related Work

The 180.1 standard used by the U.S. EPA [7] is the most widely used method to measure the turbidity of water in the laboratory. The standard can determine turbidity in drinking, ground, surface, and saline waters, as well as in domestic and industrial wastes. The application range is 0−40 nephelometric turbidity units (NTUs). Other standards have been determined to be able to specify the turbidity of water using nephelometry, of which the following four can be highlighted: ISO 7027, GLI Method 2, Hatch Method 101033, and Standard Methods 2130B [8]. Their main problem is that they are not cheap, so they cannot be used continuously for monitoring. Some authors, such as Chanson et al. [9], use acoustic waves to detect turbidity. Their method is based on backscattering, which occurs in waves when they collide with solids. Their study was implemented in an estuary in eastern Australia.

In [10], Bin Omar et al. state that turbidimeters operate based on optical phenomena. They review the standards and the different factors that can affect turbidity measurements. In addition, they described the optical properties that are important when measuring turbidity when using a fiber optic turbidimeter.

Some authors propose low-cost continuous monitoring systems for turbidity. Gillette et al. [11] present a low-cost ($64 in materials) laboratory-monitored test with a precision of 1 nephelometric turbidity unit (NTU) on a scale of 1 to 100. They performed a 38-day trial and observed a mean error between 0.45 and 1.40 NTU. Zang et al. [12] propose a low-cost turbidity sensor where they combined the extraction of spectral components (SCEs) with the signals of transmitted light (It) and scattered light (Is) and their ratio (It/Is). They used an integrated digital signal processor in combination with the Fast Fourier Transform algorithm for their implementation. Based on tests, the maximum error was 6% at 10 NTUs (turbidity), while the lowest relative error observed was 0.4% at 280 NTUs (solution). Hakim et al. [13] presented a study using the SEN0189 sensor, which measures the amount of light from the infrared LED on the phototransistor to characterize the turbidity detection capability of water. The sensor detected turbidity in the water from 1873 NTU to 1011.93 NTU. Their experiments observed that the greater the amount of dissolved sediment in the body of water, the lower the sensor output voltage. Some of the characteristics of the sensor used are the following: it has a sensitivity of −0.0008, and the output voltage when the sensor detects 0 NTUs is 3.9994 volts with an operating voltage of 5 V. Gavhane et al. [14] present a system based on LabVIEW, which allows continuous monitoring of water turbidity. The proposed system uses ARDUINO UNO for data acquisition using LabVIEW. Their findings indicate that they can monitor water quality accurately. Liu et al. [15] presented a method to control turbidity in sewage wastewater. Using their way, they managed to reduce the pollutants discharged into a river using a stormwater detention tank.

Other authors, such as Parra et al. [16], present the design, development, and calibration of a low-cost sensor capable of differentiating different sources of turbidity. They apply it in fish farms to obtain both the quantification and the characterization of turbidity. The sensor they developed is based on the Beer–Lambert law. It uses four LEDs as sources operating at different wavelengths, and the sensor is composed of a photodiode and a photoresistor located at 180° from the light sources. They created an algorithm to process the data of the samples where the turbidity, the origin, and the concentration were obtained. On the other hand, Parra et al. [17] propose a system to characterize turbidity in order to detect and track phytoplankton blooms.

Kirkey et al. [18] presented an optical backscattering transducer, which attenuates external interference from ambient light and 50 and 60 Hz light sources. Said transducer allows sensitive measurements based on low-cost circuits. They designed, built, and evaluated submersible turbidimeters based on the transducer and tested them at 7 atm pressure. The calibrated sensors had a bias of ≤0.11 NTUs in suspensions between 0 to 8 NTUs. Other authors, such as Mylvaganaru et al. [19], presented a document where they proposed the design and operation of an optical turbidity meter. It was based on measuring the re-scattered light received from illuminators that worked with light at different wavelengths. In addition, they exposed the experiment’s results in an underwater environment along with its calibration.

Some authors study turbidity in fresh water. Wei et al. [20] presented a solution to develop a low-cost turbidity sensor based on Chirp modulation and signal convolution in the statistical domain. The test system they implemented was accurate to within 2% over a range of between 0 to 100 NTUs. Takaaki et al. [21] presented a study of the monitoring and calibration of turbidimeters in the basins of the Mu and Saru rivers (Japan). The study was carried out during a period of one year at five points of the river basins. Additionally, they developed a practical estimation equation which allowed them to obtain the number of sediments in suspension from the measured turbidity. Tyler et al. [22] presented a linear mixture modeling approach to estimate chlorophyll and the concentration of suspended solids using Landsat Thematic Mapper (TM) images of Lake Balaton (Hungary).

Yaser et al. [23] propose a system in which, through the analysis of the turbidity of the water and its filtering, they obtain clean quality water, which can be reused when received from the subsoil since it is contaminated.

Authors such as Hussain et al. [24] demonstrated the possibility of measuring the turbidity of some water samples using a Smartphone. The samples used in their work were from drinking water and natural water resources. The operation of the sensor was based on the Mie scattering principle. They used two free Android apps measuring stray flow irradiance and turbidity. By varying the turbidity to 0.1 NTU, they obtained a precision ranging from 0 to 400 NTUs. They propose this turbidity detection technique as inexpensive, easy to use, and portable. Others, such as Fay et al. [25], presented a study using the Paired Emitter–Detector Diode (PEDD) technique which allowed them to measure turbidity. Taking the ISO 7027 turbidity detection standard as a reference, they compare it with other conventional photodiode–LEDs. According to their measurements, they ensure that their technique is superior to the conventional ones, and therefore they propose it is implemented in low-consumption IoT.

Authors such as Rocher et al. [26] present a wireless sensor network (WSN) that can detect sewer contamination based on solid sensors. The sensors use infrared light to see the amount of contamination in the water. They calibrated the sensors and determined a relative error of 3.4% in the range of 200–5000 mg/L. Other authors such as Tang et al. [27] propose an all-silicon photoelectric biosensor with a simple process that is integrated, miniature, and low-loss, for external monitoring. According to the authors, the all-silicon photoelectric biosensor (ASPB) has suitable characteristics for its application to handheld biosensors.

Something that distinguishes our proposal, compared to others presented by other authors, is that it is implemented at a very low cost and with very low relative errors.

## 3. Materials and Methods

This section presents the proposal description, system design, and implementation, and we explain the methodology used in the experiment.

### 3.1. Proposal Description

This subsection presents a brief description of the system requirements as well as the overall description of our system. First, we describe the hardware components. Then, we show the software structure. Finally, we propose an alarm system for malfunction detection.

#### 3.1.1. Hardware Description

The main component of the system will include the following components.

**Figure 1:**
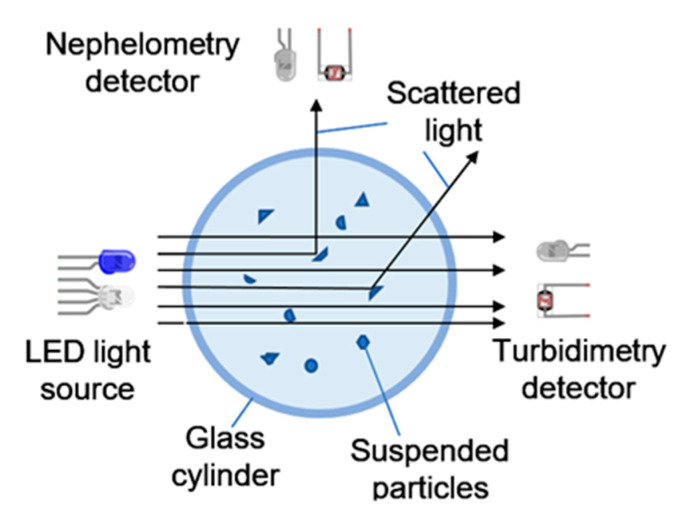
Diagram of operation of a turbidimeter and nephelometer.

**Figure 2:**
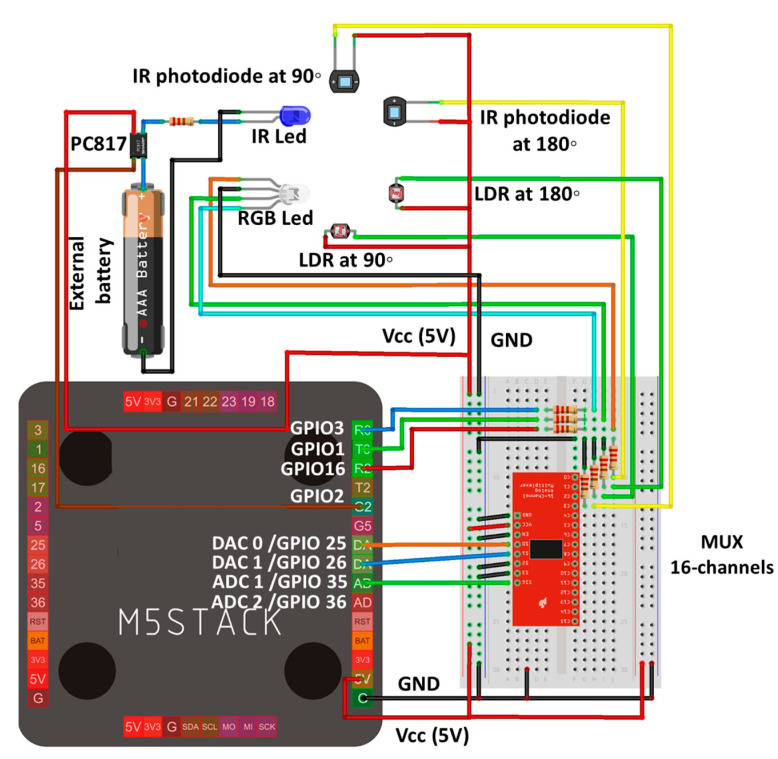
Electric scheme of the prototype.

- Microcontroller board: This was used as the central processing unit of the system, responsible for executing the software that controls the sensor. We chose M5Stack [28], a prototyping board based on the ESP32 [29] microcontroller, which provides a range of analog and digital inputs and outputs. This board was chosen for the design of our sensor due to its several advantageous features. The M5Stack board offers a color display that can be used to display the results of the sensor, as well as three buttons for operating the sensor. Additionally, it includes an SD card reader which can be used to store the results of the sensor and a small battery that allows for use of the sensor without being connected to a power source. These features make M5Stack an ideal choice for the design of our sensor. One of the drawbacks of the M5Stack is that it only includes three analog inputs, which is why it has been necessary to add a demultiplexer.
- LED light source: This was used to project the beam of light required for the nephelometry or turbidimetry measurement. We used an infrared LED (950 nm) and RGB LED to generate different wavelengths. The IR light was selected because the commercial turbidimeter uses IR light. This is because this wavelength has low energy and, therefore, will be little absorbed by the substances in the environment. The RGB wavelengths were used because the sediment and algae have different colors. Therefore, the absorption and scattering of these lights were different. Accordingly, the light that arrived at the photoreceptors was different.
- Optical sensors: We used a photodiode (1612660) for infrared light and a light-dependent resistor (LDR) for visible light. These sensors were used in pairs at angles of 90 and 180 degrees depending on whether we were using either the nephelometry or turbidimetry method. Figure 1 shows the sensors nephelometry vs. turbidimetry.

- Auxiliary elements: In order to connect the sensors and actuators to the microcontroller and adapt the different signals, we needed different resistors, as shown in Figure 2. An amplification stage was also necessary in the 90-degree infrared sensor. All these components are shown in the schematic circuit. Since the resistors have been adjusted at various times, it was thought to be more efficient to assemble the prototype on a proto board. Once the final design was reached, we then mounted the equivalent PCB board.

On the one hand, the IR LED is controlled with the pin GPIO2. In this pin, we located a resistance to limit the intensity. As the M5-Stack cannot power the LED with enough intensity, we used an external battery with an optocoupler (PC817). The pin GPIO2 powered the optocoupler to allow the current to pass (GPIO2 = 0) from the external battery to the IR LED, and we then measured with the photodiodes at 90° and 180°. On the other hand, the RGB LED has 4 terminations (3 anodes for the different colors and 1 common cathode). The pins GPIO 1, 3, and 16 were used to control the color of the LED. The different lights generated were measured with the LDR at 90° and 180°.

Finally, as the M5-stack has only 2 ADC entries (ADC1 and ADC2), we used a 16-channel MUX. This MUX was controlled with the pins GPIO 25 and 26. These pins were configurated as digital pins and allowed the measurement of 4 different entries (LDR at 90°, 180°, and photodiode at 90°, 180°). The measures were taken as the voltage drop in the terminals of the resistance elements (voltage divider). The remaining pines were saved and turned into power (VCC-5V) and a GND.

- Signal adapter circuit: To connect the sensors and actuators to the microcontroller and adapt the different signals, we needed various resistors, as shown in the circuit schematic. An amplification stage was also necessary for the 90-degree infrared sensor, for which an operational amplifier with three adjustable resistors was utilized. The M5Stack only has 3 analog inputs, but our design required 4; 2 for the infrared light sensors at 90 and 180 degrees, and 2 for the visible light sensors. To overcome this issue, we added a multiplexer which can select up to sixteen inputs in one process.

All of these components are shown in the circuit schematic. As the resistors had been adjusted at various times, it was more efficient to assemble the prototype on a protoboard. Once the final design was achieved, we mounted the equivalent PCB board.

**Figure 3:**
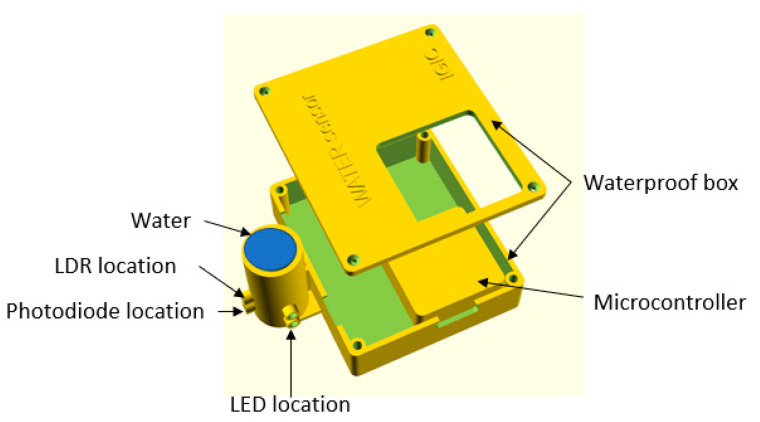
Scheme waterproof box.

- Sensor Enclosure: To protect the sensor and its components, we designed and 3D printed an enclosure for the sensor. Having a protective case for the sensor reduced the risk of cable disconnections that can occur when using a proto board, as well as protecting the components from water damage. Additionally, it allowed for the precise positioning of the LEDs. Figure 3 shows the sensor.

The sensor enclosure is composed of two compartments. The larger compartment houses the M5Stack and the signal adaptation circuits, including a multiplexer, operational amplifier, resistors, and capacitors. This compartment has a cover with a window that provides access to the M5Stack screen and buttons. The second compartment is a cylindrical container that holds a glass container with a diameter of 25 mm for the liquid to be measured. The cylinder has openings to place the light emitters and receivers at 180 and 90 degrees. These openings are located at two different heights, with the infrared emitters/receivers at 10 mm from the base and the visible light ones at 20 mm.

#### 3.1.2. Software Functionality

The software program was responsible for controlling the operation of the sensor and collecting the data generated by the measurements. The program included a user interface that allowed the user to configure the measurement parameters, such as the refresh_time and samples_number. They could also be configured if we wanted a graphical representation of the outputs on the m5stack screen. Algorithm 1 shows the Master head algorithm.

Once the configuration was completed, a loop was performed in which 9 phases were passed through. In each phase, a different LED light source and photosensor were activated. The names of the phases were “IR180”, “IR90”, “R180”, “R90”, “G180”, “G90”, “B180”, “B90”, “NONE”. In the first two phases, infrared emitters and receivers were used, first at 180° using the turbidimetry technique and then at 90° using the nephelometry technique. During the next 6 phases, visible light was used in red, green, and blue frequencies. Measurements were taken at 180° and 90° for each color. The last phase was used in the malfunction detection system, which is described in the next section.

In each phase, several readings were taken at equal time intervals. These values were set in the algorithm’s parameters samples_number and refresh_time (samples_number = 320 and refresh_time = 10 ms). In the case of IR90, a much larger samples_number was used, since the sample was noisy and therefore we wanted to make a longer average, taking up to 40 s. Every time a phase was finished, the mean and standard deviation of the samples were obtained.

When the 9 phases were completed, all the information obtained was stored on the M5Stack’s SD card. To do this, a new line was added to a text file, which included the date and time the sample was taken, the mean and variance values obtained in the first 8 phases, and information about the malfunction detection phase. This file can be used for statistical analysis of the data and for calibrating the device.
**Algorithm 1:** Master head algorithm.Given: refresh_time, samples_number PHASE=[“IR180”,“IR90”,“R180”,“R90”,“G180”,“G90”,“B180”,“B90”,“NONE”] configure_graphical_output_UI() configure_pinMode_for_each_in_out() Repeat    For Each phase In PHASE      select_multiplexer_input(phase)     turn_on_appropriate_LED(phase)     For Each count In 1..samples_number      value = read_analog_value()      save_value(phase, count, value) // o data[phase,count] = value      draw_value_UI(value)      wait(refresh_time)    End For     obtain_mean_and_deviation(phase)  End For   check_alarm_system()   save_phases_in_SD() Until button_C is pressed

To ensure the reliability of the measurement data, we included a system for detecting malfunctions in the sensor. This system consisted of sensors that detected any changes in the operating conditions of the sensor. The alarm alerted the user to any potential problems with the sensor, allowing them to take corrective action before the measurement data were compromised.

For the first 8 phases of the sensor, a maximum and minimum acceptable value was defined, both for the mean and for the variance. If any phase did not obtain values within its range, the corresponding alarm would be displayed to the user.

To prevent possible contamination by parasitic lights, a ninth phase was added where the photosensors were read without activating any LED. In these cases, the signal read needed to be 0, or very close to 0.

### 3.2. Test Bench

In this subsection, we explain the methodology used in the experiment.

#### Methodology

In this subsection, we present the methodology used to obtain the different results. The measurements were performed in laboratory conditions, with a temperature of 25 °C and 1 atm of precision. The methodology employed was the same as that presented by Rocher et al. [26]

**Figure 4:**
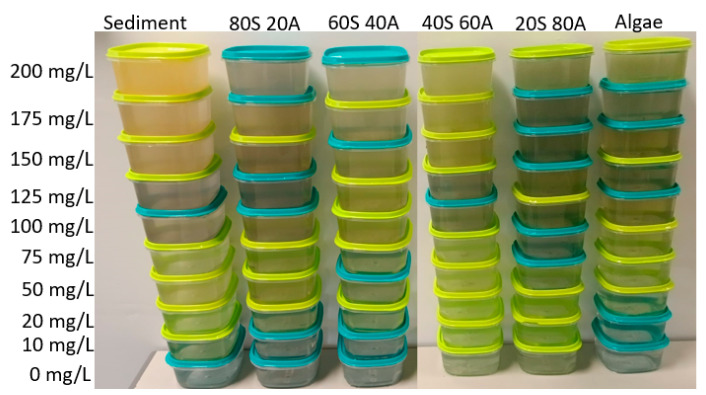
Samples process.

First, we explain the elaboration of the different samples used in the experiments. We tested with different solid concentrations. The solids were obtained from the dilution of clay and algae. The concentrations tested were 0, 10, 20, 50, 75, 100, 125, 150, 175, and 200 mg/L; each concentration was tested with different percentages of algae and solids. The different percentages of algae or clay tested were 0, 20, 40, 60, 80, and 100%. In total, 55 samples have been performed, and these can be observed in Figure 4. We named these mixtures using the acronyms of the algae (20S 80A, 40S 60A, 60S 40A, and 80S 20A) and sediment. To obtain the stock solutions, we diluted 40 g of clay in 1 L of water and 50 mg/L of algae in 1 L of water. This dilution was left to settle for 4 h to eliminate those impurities that could cause the samples to be inhomogeneous. We obtained two solutions, one with 18.53 g/L of clay and the other with 40.12 g/L of algae. These two solutions were diluted to process the different samples.

A commercial turbidimeter model TU-2016 [30] was used to measure the turbidity of the samples. We used two standard calibration samples of 0 and 100 NTUs, and the measures were performed with 10 mL of the sample and using the glasses that included the turbidimeter. The values obtained with it were compared with the results obtained with our prototype. In our prototype, we can differentiate two types of light (visible and infrared (IR)). We used one IR LED and two photodiodes at 90° and 180°. On the one hand, we used an IR LED (TSUS5400 model), which features a diameter of 5 mm, peak wavelength of 950 mm, and a forward current of 150 mA [31]. In visible light, we used a RGB LED model (L-154A4SUREQBFZGEW), which features a diameter of 5 mm [32]. On the other hand, as photoreceptors, we used two OSRAM photodiodes (SFH 203 model), which feature: a switching time of 5 ns, a diameter of 5 mm, and a package in clear epoxy [33]; we also used an LDR model NSL-19M51 [34]. The photodiode detected the IR light and the LDR detected the visible light. Finally, we used a glass that contained the sample inside. The diameter of the glass was 2.5 cm, and the height was 7.8 cm.

With the different samples elaborated, we measured the turbidity in triplicate with the commercial turbidimeter calibrated with commercial samples of 0 and 100 NTUs. The results obtained with the commercial turbidimeter were compared with the response of our prototype.

To power the prototype, we used the M5Stack and an external battery to power different circuits. We opted for using this power source versus other alternatives, such as a power supply, because the prototype was in nature, where access to an external power source was impossible. The external battery was used to power the IR LEDs. The RGB LED, photodiodes, and LDRs were powered with the M5stack. The LDRs were powered with 3.3 V, and the other elements were powered with 5 V. To prevent the effect of the external light when we collected the measures, we covered the prototype with an opaque plastic box.

The photoreceptor circuits were based on a voltage divider formed by the photoreceptor and a fixed resistor. The use of semiconductors based on silicon with p–n junction (LEDs) has generated important uses in recent years [35]. On the one hand, to determine the resistance in the LDR circuits, first, we measured the resistance with a multimeter at 0 and 200 mg/L of clay, powering the colors red, blue, and green at 90° and 180°. With the values of resistance and Equation (1) (voltage divider formula), we calculated the resistance that maximized the voltage differences between the different lights. In Equation (1), the Vout is the voltage that was read by the microcontroller, Vin is the voltage powered in the voltage divider (3.3 V), R_LDR_ is the resistance of the LDR, and R_fix_ is the resistance selected. On the other hand, with the photodiode, we tested with the resistance shown in Table 1. Using the resistances used in the IR LEDs (controlling the intensity and luminosity) and the resistance in the voltage divider circuit of photodiodes, we tested the different possible combinations to select the better configuration of resistances. The samples used in this test were 0, 100, and 200 mg/L of clay. We selected these to find the higher voltage difference in the range that we wanted to measure. In addition, we used the sample of 100 mg/L to know if the voltage difference was produced in the higher concentrations tested or in the lower concentration.

(1) $$Vout=Vin*\frac{R_{LDR}}{R_{LDR}+R_{fix}}$$

Once the resistances for the different circuits were selected, the different samples were tested. The samples were introduced into the glass and the glass into the prototype to obtain the data, the values of the selected configurations were obtained, and the sample was withdrawn. This process was carried out in triplicate. The lights used were IR, red, green, and blue that light up sequentially, and the measurements were collected at 180° and 90°.

## 4. Results

In this section, we analyze and discuss the results obtained by our prototype. First, we select the better combination of resistance. Then, we analyze the results obtained. Finally, we analyze the mathematical model related to turbidity with the sensor response.

### 4.1. Selection of the Better Combination of Resistance

**Figure 5:**
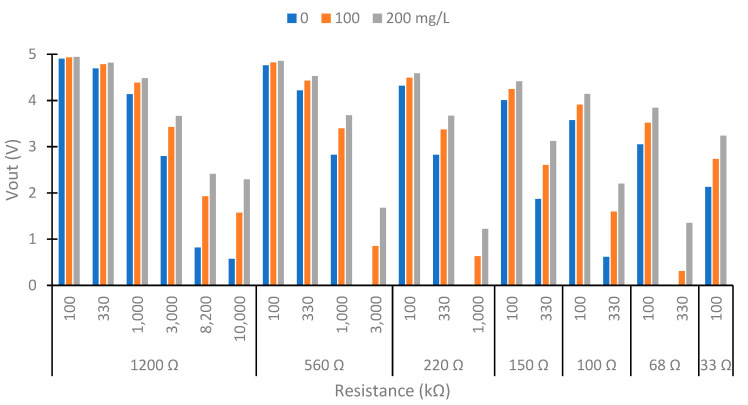
Test of the configuration of IR circuits. LED resistance 1200 Ω, 560 Ω, 220 Ω, and 150 Ω at 180°.

In this subsection, we show the different combinations of currents to power the IR LED and resistances in the voltage divider of the photodiodes. We perform the test with three concentrations: 0, 100, and 200 mg/L. Figure 5 shows the Vout obtained at 180° higher than 0 V. In the configurations not represented in Figure 5, the Vout is 0 in the three samples. That indicated that the photodiode’s resistance is much lower than the circuit’s fixed resistance. These configurations are unsuitable for our prototype because there are no differences between the samples. In the configuration that is suitable, we observe that when the current in the IR LED increases, the Vout decreases. The same occurs when we increase the resistance in the voltage divider. This occurs because the resistance difference between the photodiode and the fixed resistor is vast. What causes all of the voltage drop occurs in the resistance. Therefore, too much light would reach the photodiode for the selected resistance in these cases. The maximum voltage registered is 5 V, the maximum M5stack can read. In our prototype, we are interested in maximizing the voltage difference between the minimum and maximum solid concentration to have maximum sensibility. The maximum voltage difference is in using a resistance of 1200 Ω in the IR LED and 10 MΩ in the voltage divider with a value of 1.713 V. The values obtained are 0.576, 1.571, and 2.289 V in concentrations of 0, 100, and 200 mg/L, respectively. The second maximum difference is 560 Ω in the IR LED configuration and 3 MΩ in the voltage divider with a difference of Vout of 1.678 V. The voltages obtained in 0, 100, and 200 mg/L are 0, 0.855, and 1.678 V, respectively. 

**Figure 6:**
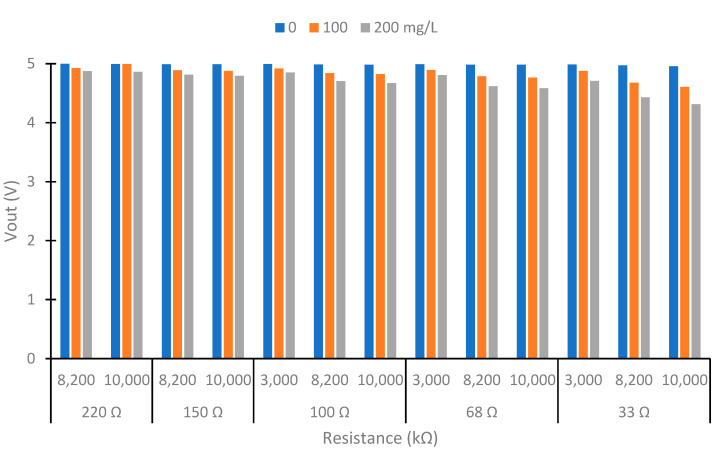
Test of the configuration of IR circuits. LED resistance 150 Ω, 100 Ω, 68 Ω, and 33 Ω at 90°.

Now, we analyze the results to select the better configuration in the photodiode at 90°. Figure 6 shows the results obtained in the photodiode at 90° with configurations with a Vout difference between 0 to 200 mg/L higher than 0.1 V. The other configurations are unsuitable in our prototype. The Vout in the configuration not represented is 5 V or near its value. This indicates that little light reaches the photodiode, so its resistance is higher than the fixed resistance, and most of the voltage drop occurs in it. The response of the IR at 90° is the opposite of that observed at 180°. The response of the IR at 90° is the opposite of that observed at 180°. This occurs because as solids increase, less light can cross the water, and more light is scattered in all directions. The increase in solids decreases the Vout in the photodiode at 90°. However, the opposite effect would occur if the solids’ concentrations were too high. Once a particle scattered the light, it would collide with another particle, reducing the dispersion.

In this use of the photodiode at 90°, with a concentration of 0 mg/L, the Vout is near 5 V. This happens because the water without solids does not disperse much of the light. Then, the resistance of the photodiode is high. The minimum voltage obtained is 4.314 V with a concentration of 200 mg/L and with the maximum resistance in the voltage divider and intensity of the LED. This value is far from 0 V. This indicates that the resistance in the photodiode is bigger than the fixed resistance. So, the use of a more sensitive photodetector and a light emitter with more powerful light emitters can be studied in future work to improve the sensor’s sensitivity. We select the LED with 33 Ω and 10 MΩ in the voltage divider as the configuration of power, as it is the configuration with the greatest voltage difference (and therefore greater sensitivity).

The IR LED and voltage divider circuit configuration were selected. We determined the resistance values used in the voltage divider circuits of the LDRs at 90° and 180°. Table 2 represents the resistance values obtained, the fixed resistance used, and the voltage. As expected, there existed a proportional relation between the resistance of the LDR and the solid concentration at 180°, which was inversely proportional at 90°. This was caused by the increase in solids, reducing the light that could cross the water but increasing the light scattering. We observed that the resistance values were higher in the LDRs at 90° than 180°. However, the resistances of 90° were similar between them, as was the case with 180°. With the resistance values, we calculated the ideal resistance for each light with Equation (1). As in our prototype, we could not locate six different circuits; we needed to select two resistances (LDR at 180° and 90°). The selected resistances were 1.2 kΩ in the voltage divider at 180° and 33 kΩ at 90°. The difference between the optimal and select resistance was minimal (0.011 V in the case with more difference).

If we observe the values of voltage difference in the case of the voltage divider of the LDR at 180°, the differences are less than 0.3 V. These differences are lower in comparison to those obtained in the LDR at 90°. The differences are similar to the blue and green light, with values near 0.674 V. Nevertheless, the difference is 1.276 V for red light. The sediment causes the light used to be red and, therefore, will scatter more light of this color.

In our prototype, we tested the configurations of 560 Ω and 1200 Ω in the IR LED and 3 MΩ and 10 MΩ in the voltage divider photodiode at 180°, respectively. At 90°, we use 33 Ω in the IR LED and 10 MΩ in the voltage divider. In the RGB LED, we select 1.2 and 33 kΩ as fixed resistances in the voltage divider at 180° and 90°, respectively.

### 4.2. Turbidity of Samples

In this section, we analyze the turbidity of the samples elaborated. We use commercial turbidity to determine the turbidity values.

**Figure 7:**
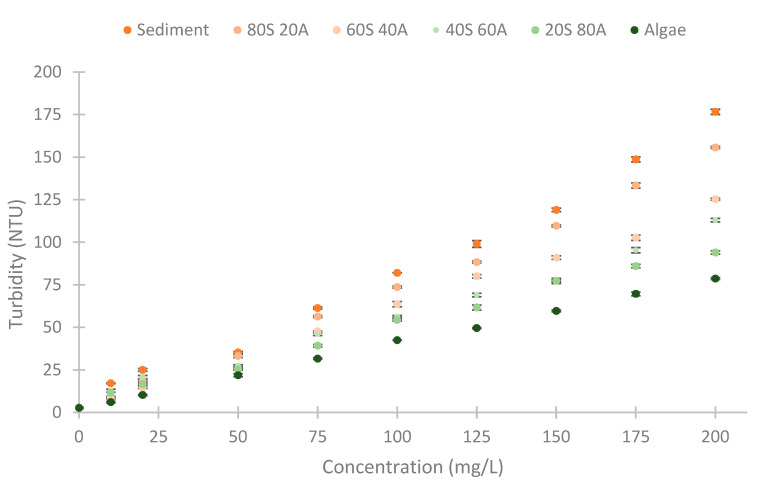
The turbidity of the different samples measured with a commercial turbidimeter.

The data gathered are represented in Figure 7. We observe that the NTUs of the samples elaborated with sediment (clay) have more turbidity than the algae samples. The different mixtures of algae and sediment have turbidity between the pure samples. If we study the relation between turbidity and concentration, there is a linear relation between the concentration of the solid and the turbidity of each tested mixture. The R^2^ values of these correlations are 0.9888, 0.9955, 0.9950, 0.9912, 0.9943, and 0.9994 for the samples: sediment, 80S 20A, 60S 40A, 40S 60A, 20S 80A, and algae, respectively. These high correlations indicate a strong correlation between the variables’ turbidity and solid concentrations for the different percentages of algae and sediment. The linear slope is useful to monitor all of the range because it indicates differences between the different concentrations of solids and turbidity. The maximum turbidity registered is 176.7 NTUs in the pure sediment and 200 mg/L of solid concentration. The standard deviation of the samples is slight. The maximum standard deviation is 2.06 NTUs in the sample of 10 mg/L mixture 20S 80A, and the mean turbidity of this sample is 11.7 NTUs. The mean of the standard deviation of all samples tested is 0.78 NTUs. Regarding its distribution, there does not seem to be a pattern that relates the standard deviation values to the turbidity values recorded.

With the values of the turbidity of the different samples performed, we then tested the different RGB and IR LEDs.

### 4.3. IR LED

In this subsection, we analyze the results obtained with the IR light. The resistances used in the photodiode voltage divider circuit and the intensity of the IR LEDs are selected.

**Figure 8:**
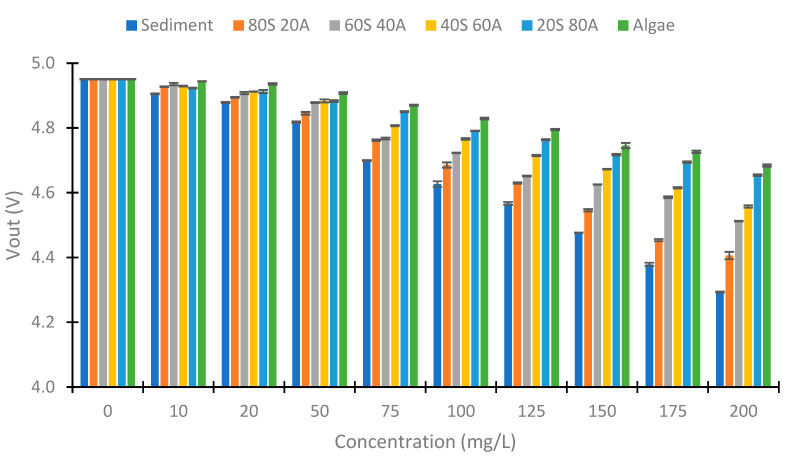
Vout in the voltage divider of the photodiode at 90°.

Figure 8 represents the Vout obtained using IR light in the photodiode at 90°. We can observe a decrease in the Vout with the increase in the solid concentration. However, the decrease is less with an increase in the algae percentage. This occurs because the solids from the algae carry less turbidity than the sediment solids, as we have previously seen in Figure 7. The voltage differences between the concentrations of 0 to 200 mg/L are 0.658, 0.545, 0.439, 0.393, 0.296, and 0.267 V in relation to the samples sediment, 80S 20A, 60S 40A, 40S 60A, 20S 80A, and algae, respectively. The differences between the Vout and the % of algae in the sample are more evident with the increase in the solid concentration. In the concentration of 10 mg/L, the Vout of the samples with 80, 60, 40, and 20% sediment is near 4.929 V. However, the sediment sample has a Vout of 4.906, and the sample without sediment has a Vout of 4.944 V. In the concentration of 20 mg/L, there exists an increase in the Vout with an increase in the % of algae, except in the samples with 40 and 20% of sediment and that have similar values of Vout. In the higher concentration of 50 mg/L, the increase in Vout with the % of algae is evident in all concentrations. Finally, in terms of the standard deviation, this is small. The maximum standard deviation is 0.01 V. Finally, the decrease in Vout in the different samples is linear with the turbidity, with R^2^ values of 0.9958, 0.9975, 0.996, 0.9956, 0.9971, and 0.9889 in relation to the samples of sediment, 80S 20A, 60S 40A, 40S 60A, 20S 80A, and algae, respectively. This is indicative of a strong correlation between turbidity and the Vout.

**Figure 9:**
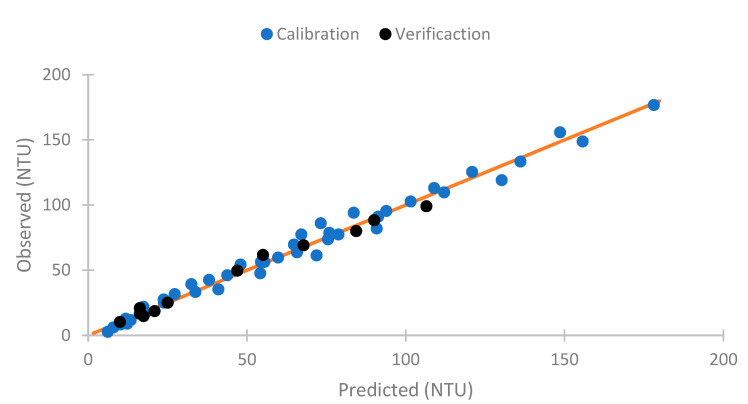
Observed versus predicted NTU values model.

As the results obtained respond to the turbidity, we perform a model to compare the sensor response with the turbidity of the samples. To perform the model, we use the data gathered, except the concentrations of 20 and 125 mg/L. These are used for verification. Equation (2) is this mathematical model. This is a linear model with a R^2^ of 0.983. Thus, there is a strong correlation between the observed and predicted values. The observed and predictive values of this model are represented in Figure 9. In this figure, we can see that the predicted values are like the observed ones. In general, the values observed and predicted trends are similar. The model’s residuals are low if we observe the orange line (observed and predicted values are equal). For this reason, our model is good for predicting the NTU figure. However, the model has deficiencies in determining the sample’s turbidity with 0 mg/L of solids (2.73 NTUs). In this concentration, the absolute error is 3.47 NTUs, representing a relative error of 127.42%. If this concentration is eliminated, the mean calibration error is 4.04 NTUs, and the relative error is 8.90%. In the verification, the errors are 2.85 NTUs and 7.45%. This is similar to the commercial turbidity used, which has an error of 5% in the measure (or 0.5 NTUs).

(2) $$Turbidity\;\left(NTU\right)=1300.66-261.451*\mathrm{Vout}\;\left(V\right)$$

**Figure 10:**
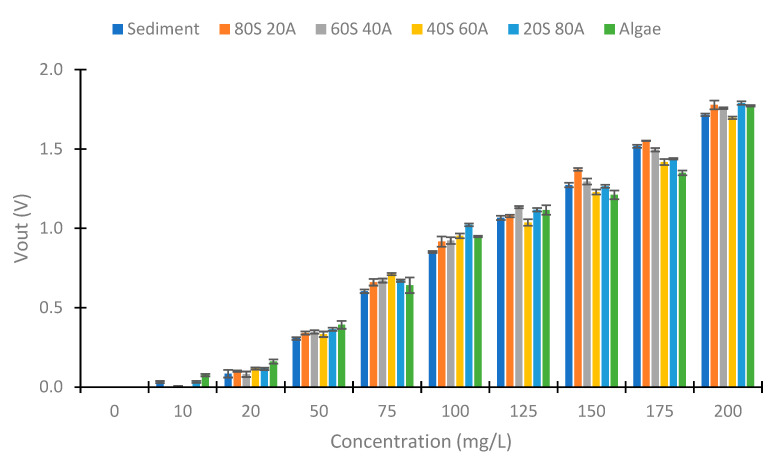
Vout in the voltage divider of the photodiode at 180°, with 560 Ω and 3 MΩ.

**Figure 11:**
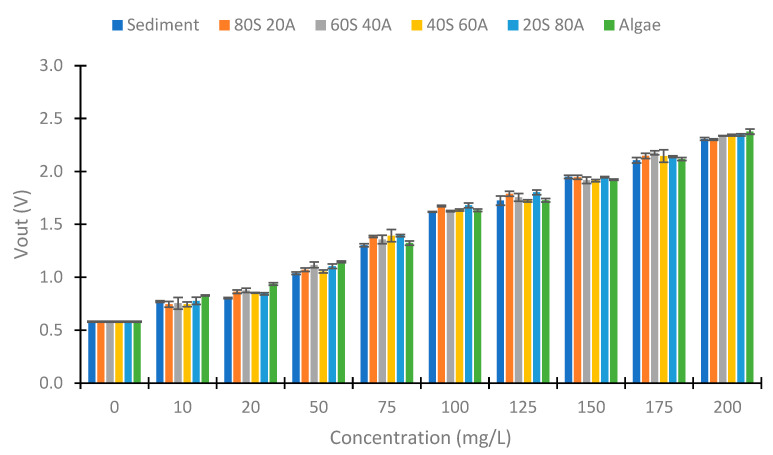
Vout in the voltage divider of the photodiode at 180°, with 1200 Ω and 10 MΩ.

Once the data obtained with infrared light at 90° have been analyzed, we proceed to analyze the data obtained at 180°. Figure 10 and Figure 11 represent the Vout obtained at 180° in the two configurations tested. Contrary to the previous case (IR at 90°), there is not an evident trend of voltage change with increasing algae in the samples. This is because, in the photoreceptors at 180°, light absorption is the most important phenomenon. At 180°, the Beer–Lambert law can be applicated. According to this law, light absorption is proportional to the absorbance constant (depending on the substance), the distance that the light travels, and the concentration of the substance. Meanwhile, at 90°, the backscattering of the light is the essential effect. The backscattering depends on different effects, such as the Tyndall effect and Rayleigh scattering, among others. Figure 10 represents the Vout obtained with the configuration of 560 Ω in the IR LED and 3000 kΩ in the voltage divider. We observe an increase in the Vout with the increase in the solid concentration. The minimal Vout observed is 0 V in the 0 mg/L concentration. The difference in voltage between the minimal Vout (in 0 mg/L) and maximum Vout (in 200 mg/L) is 1.79 V. With respect to the evolution of the decrease in the Vout, the concentrations of 0 and 10 mg/L present similar values (near 0 V). In the concentration of 20 mg/L, the Vouts obtained are around 0.100 V. From this concentration, there is a proportional decrease between the voltage and the concentration of solids. Figure 11 shows the Vout of another configuration tested in the IR light at 180°. In this case, the voltage reduction occurs in all the concentrations tested with a linear decrease. For this configuration, the maximum Vout is 2.377 V, and the minimum is 0.581 V, which implies a difference of 1.796 V. In this configuration, we observe a difference of Vout in all concentrations. In addition, for the same concentration of solids, the values are more similar to those observed in the other configuration.

With the data observed, the IR light at 180° of the photoreceptors can be used to monitor the concentrations of solids. Equation (3) represents the values obtained by using the configuration of 560 Ω in the IR LED and 3000 kΩ. This model has a R^2^ of 0.990, and Equation (4) represents the configuration of 1200 Ω in the IR LED and 10 MΩ and has a R^2^ of 0.994. Table 3 represents the errors obtained by using the models. We can observe that the mean absolute error in the two configurations is similar—5.69 mg/L in model 3 and 4.53 mg/L in model 4—to the calibration values. The relative errors are 11.15 and 10.73% for models 3 and 4, respectively. The verification errors are minor in model 3, with a value of 3.91 mg/L as an absolute error and a relative error of 11.84% in front of 4.46 mg/L and 12.63% of the other model. We select 1200 Ω in the IR LED and 10 MΩ. Because the differences are minimal, this will allow the system to be improved in the future by using two IR LEDs at 180° and 90°, which allows for saving in terms of the number of analogue inputs of the microcontroller.

Concentration (mg/L) = 4.30241 + 112.413 * Vout (V)(3)

Concentration (mg/L) = (5.657 + 9.897 * Ln(Vout (V))^2^ (4)

In summary, on the one hand, using the IR light at 90° helps determine the turbidity. In our case, the voltage difference between the minimal and maximum value registered is 0.658 V, and the error in the measure is 8.9% in the calibration and 7.45% in the verification. On the other hand, using the IR light at 180° is useful to determine the solid concentration. The voltage difference is 1.796 V, and the error is 8.09% in the calibration and 11.40% in the verification. With these two parameters, we can determine the % of algae in our experiment, because for the same concentration of algae or sediment, the turbidity varies. However, this will not be viable in more complex environments, so it is necessary to use other wavelengths of light for its determination.

### 4.4. Use of Colour LEDs

In this subsection, we analyze the results obtained using the RGB LED at 180° and 90°.

**Figure 12:**
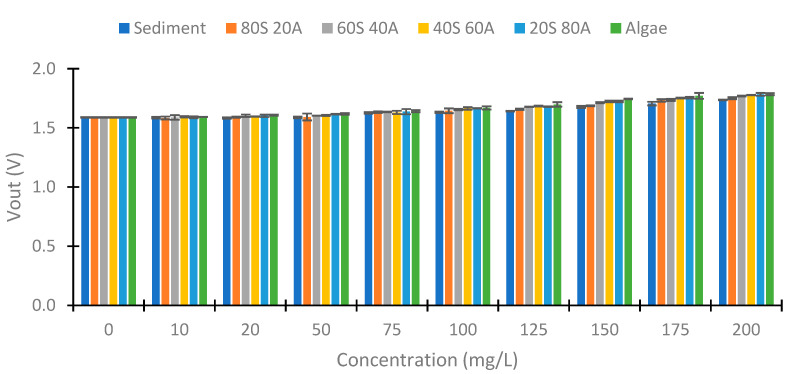
Vout with the use of red light at voltage divider LDR at 180°.

**Figure 13:**
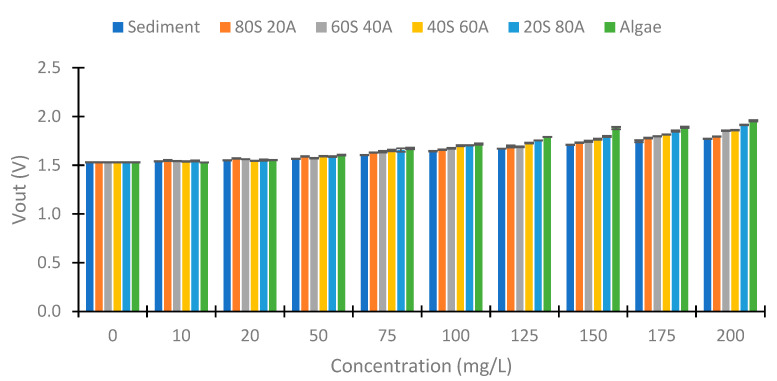
Vout with the use of green light at voltage divider LDR at 180°.

**Figure 14:**
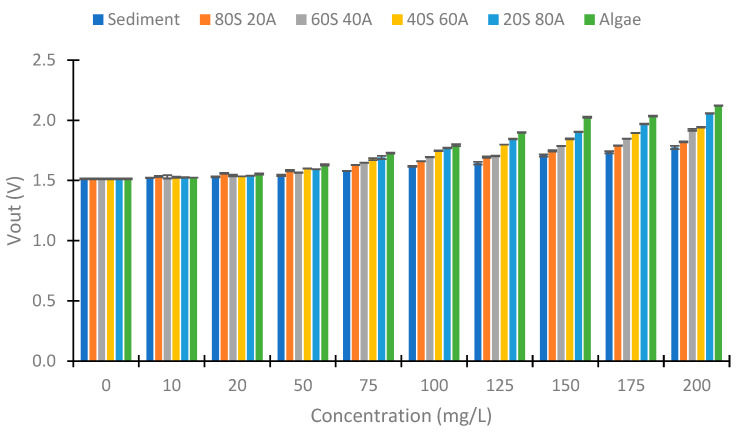
Vout with the use of blue light at voltage divider LDR at 180°.

First, we examine the values obtained at 180°. Figure 12 shows the values obtained with red light in the voltage divider at 180°. We can observe a voltage increase between the 50 to 200 mg/L concentrations in this case. The minimum voltage obtained is 1.583 V, and the maximum Vout is 1.785 V (Algae 200 mg/L). This implies a voltage difference of 0.203 V. Figure 13 represents the values obtained with the use of green light. In this case, the difference between the minimal and maximum voltage registered is 0.428 V. Figure 14 represents the values obtained using blue light. In this case, the maximum difference is 0.608 V, produced in the samples with 100% algae. The values between 0 and 50 mg/L are similar in the three cases. From the concentration of 75 mg/L, an increase in Vout begins to be observed. Contrary to the case of the infrared light at 180°, a difference in Vout with the increase in the % of algae is observed. This indicates that the algae absorb more light than the sediment (algae have a greater absorption coefficient than sediment).

**Figure 15:**
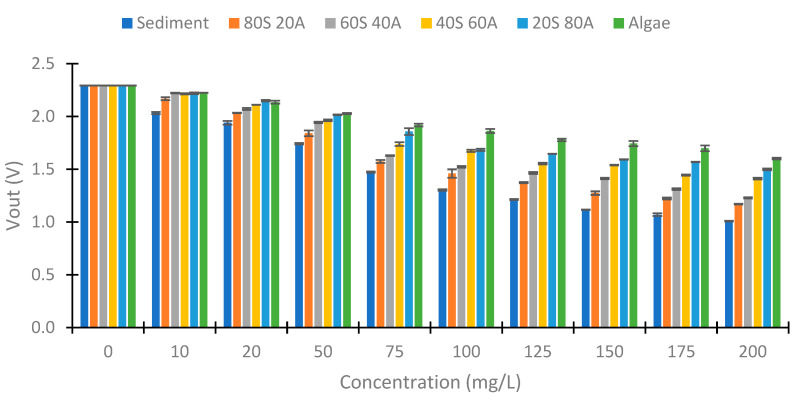
Vout with the use of red light at voltage divider LDR at 90°.

As in the IR light, we tested with different angles of the photoreceptor in the RGB light. Figure 15 represents the values obtained with the use of red light. As in the case of the IR at 90°, we observed a decrease in the Vout with an increase in the solid concentration, but this decrease is less with the increase in the % of algae. In the sample with sediment, the Vout difference is 1.284 V and in the algae, it is 0.693 V. In the concentration of 10 mg/L, the samples of sediment and 80S 20A have Vout values different than the other samples, 2.033 and 2.169 V, respectively. In the other samples, the values are near 2.220 V. In the concentration of 20 mg/L, the samples 20S 80A and algae have similar Vout (1.615 V). In the concentrations higher than 50 mg/L the Vout difference in relation to the percentage of algae is more evident.

**Figure 16:**
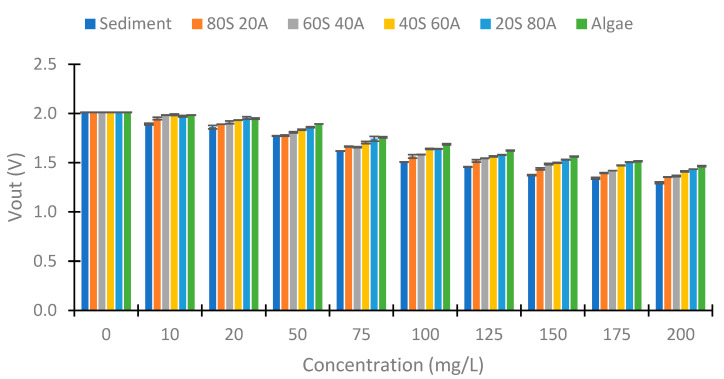
Vout with the use of green light at voltage divider LDR at 90°.

Another light used is green. Figure 16 shows the Vout obtained with this light. In this case, the differences are less than with the use of red light. In the sediment, the Vout difference is 0.715 V in sediment and 0.548 V in algae. In this light, the change in Vout in the same concentration but the different percentages of algae are less than in red light. As in the previous case, the differences between the different percentage of algae are more evident with the increase in the solid concentration.

**Figure 17:**
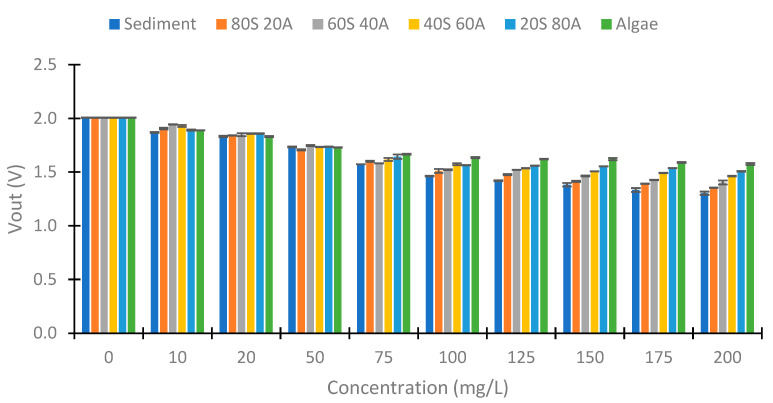
Vout with the use of blue light at voltage divider LDR at 90°.

Finally, in Figure 17 are represented the Vout values using blue light. In the samples with 100% sediment, the Vout difference between 0 to 200 mg/L is 0.703 V (a voltage difference similar to that obtained with green light). However, in the sample without sediment, the Vout difference is 0.431, more than 0.1 V of difference in comparison to green light. This indicates a different behavior compared to what was observed with the previous green visible light. The difference in Vout in the different percentages of algae can be observed in concentrations equal to or greater than 75 mg/L. In the concentrations that are less, there are no differences in the Vout. In the concentration of 10 mg/L, the values are between 1.943 and 1.870 V without following a distribution according to the % of algae. In 20 mg/L, the Vout is between 1.830 to 1.859 V, and in 50 mg/L, the Vout is between 1.708 to 1.747 V.

With the Eureqa software [36], we calculated the models that related the algae concentration in water with the sensor’s response. As with infrared light, 20 and 125 mg/L concentrations are used for model verification. The Eureqa software offers different solutions, as the more complex the model is, the better the prediction of the algae concentration, but a calculation with more capacity needs the microcontroller. First, the mathematical models are calculated with the Vout of 90° visible light data, and the turbidity and concentration values are calculated with Equations (2) and (4). Equations (5) and (6) are then obtained. In the mathematical models, V_outR_ is the Vout with the red light at 90°. V_outG_ is the Vout with the use of the green light at 90°. V_outB_ is the Vout with the use of blue light at 90°. The R^2^ of the models are 0.992 in relation to Equation (5) and 0.988 in relation to Equation (6). The errors of these models are represented in Table 4. Equation (5) has fewer errors in calibration and verification than Equation (6). The absolute errors are 5.73 mg/L in the most complex model against 6.85 mg/L in the other model. These values represent relative errors of 19.17% and 29.49%. In the verification, the absolute errors of Equation F are higher in terms of sensibility than in the calibration, especially in the verification with a relative error of 53.33%. As Equation (6) does not present a much greater calculation complexity, it is advisable to use this equation over the other.


(5) $$\mathrm{Algae}\left(\frac{\mathrm{mg}}{L}\right)=158.697*V_{\mathrm{outR}}\left(V\right)+0.441*\mathrm{Concentration}\left(\frac{\mathrm{mg}}{L}\right)+0.000837*\;V_{\mathrm{outB}}\left(V\right)*0.00133^{V_{\mathrm{outR}}\left(V\right)}*{\mathrm{Concentration}}^{3\;}\left(\frac{\mathrm{mg}}{L}\right)+0.000837*V_{\mathrm{outB}}\left(V\right)*\;{\mathrm{Concentración}}^{2}\left(\frac{\mathrm{mg}}{L}\right)*V_{\mathrm{outR}}{}^{2}\left(V\right)-33.0381-0.00133^{\mathrm{VoutR}\left(V\right)}-0.753*\;\mathrm{Turbidity}\;\left(\mathrm{NTU}\right)-81.471*V_{\mathrm{outG}}\left(V\right)*V_{\mathrm{outB}}\left(V\right)$$

(6) $$\mathrm{Algae}\left(\frac{\mathrm{mg}}{L}\right)=127.053+169.956*V_{\mathrm{outR}}\left(V\right)+0.683*\mathrm{Concentration}\left(\frac{\mathrm{mg}}{L}\right)*\;V_{\mathrm{outR}}\left(V\right)-255.616*V_{\mathrm{outG}}\left(V\right)-0.469*\mathrm{Turbidity}\;\left(\mathrm{NTU}\right)*V_{\mathrm{outG}}\left(V\right)$$

Another option is using all the data collected (90° and 180°). In this case, we obtain Equation (7) with a R^2^ of 0.994 and Equation (8) with a R^2^ of 0.993. In the mathematical models, V_outR180_ is the Vout with the red light at 180°. V_outG180_ is the Vout with the use of the green light at 90°. V_outB180_ is the Vout using the blue light at 90°. The two models have similar values of R^2^. However, as shown in Table 5, the absolute errors of the two models in the calibration are 3.74 and 3.99 mg/L of the algae in Equations (7) and (8), respectively. Nevertheless, the relative errors are higher in Equation 7 than in Equation (8). In Equation (7), the relative error is 14.98%, and in Equation 8, it is 11.61%. In the verification, the opposite occurs. The errors of Equation (7) are smaller than those of Equation (8). For this reason, we use Equation (7). With all the data, the errors are more minor than using only those that are of 90°. That is why the elimination of data should not be considered if the intention is to develop a mathematical model that relates the sensor’s response to the algae concentration.


(7) $$\mathrm{Algae}\left(\frac{\mathrm{mg}}{L}\right)=331.641*V_{\mathrm{outB}180}\left(V\right)+219.442*V_{\mathrm{outR}180}\left(V\right)+36.554*V_{\mathrm{outG}}\left(V\right)*\;V_{\mathrm{outB}180}\left(V\right)+0.105*\mathrm{Concentration}\left(\frac{\mathrm{mg}}{L}\right)*V_{\mathrm{outR}}\left(V\right)*V_{\mathrm{outG}}\left(V\right)*V_{\mathrm{outB}180}\left(V\right)\;-515.187-291.554*V_{\mathrm{outG}180}\left(V\right)-0.220*\mathrm{Turbidity}\;\left(\mathrm{NTU}\right)*V_{\mathrm{outB}180}\left(V\right)$$

(8) $$\mathrm{Algae}\left(\frac{\mathrm{mg}}{L}\right)=168.469*V_{\mathrm{outB}180}\left(V\right)+73.716*V_{\mathrm{outG}}\left(V\right)*V_{\mathrm{outB}180}\left(V\right)+0.359*\;\mathrm{Concentration}\left(\frac{\mathrm{mg}}{L}\right)*V_{\mathrm{outR}}\left(V\right)-478.582-0.354*\mathrm{Turbidity}\;\left(\mathrm{NTU}\right)$$

**Figure 18:**
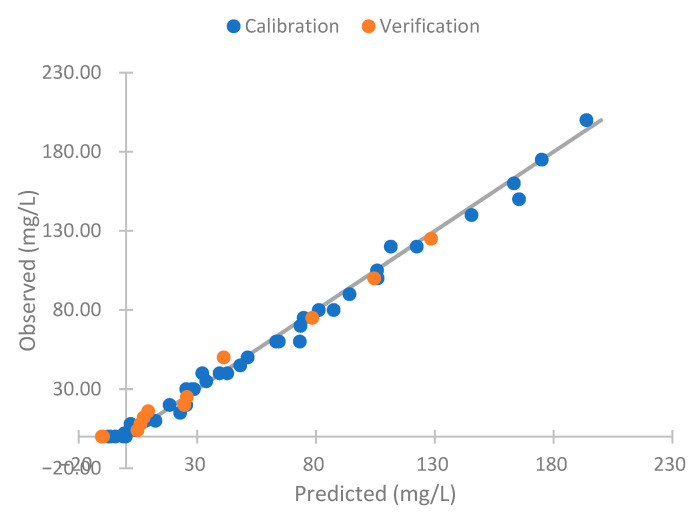
Observed vs. predicted Equation (7).

**Figure 19:**
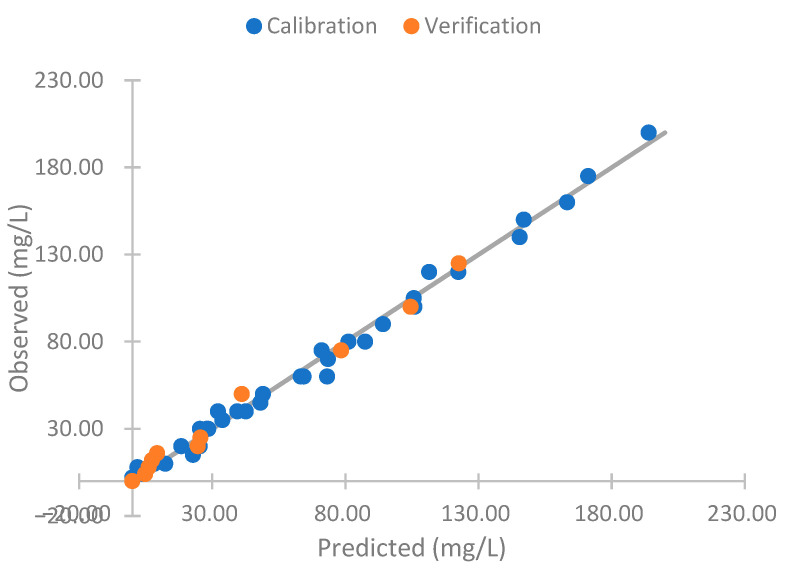
Observed vs. predicted Equation (7) improved.

The observed versus predicted values of Equation (7) are represented in Figure 18. We can observe that the model adequately predicts the observed values of the algae concentrations. However, the model presents a negative algae concentration. This is impossible in real conditions. Thus, we can apply four conditions to improve the model’s performance: (I) The NTU reading cannot be negative; (II) the solid concentration cannot have negative values; (III) the concentration of algae cannot be negative; and (IV) the algae concentration cannot be higher than the solid concentration. Thus, we obtain Figure 19. These conditions do not present negative concentrations. The errors of these modifications of the model are shown in Table 6. The absolute error is 2.78 mg/L, and the relative error is 13.91 in the calibration. With this, we obtain a reduction of approximately 1 mg/L of algae in the calibration error and a reduction of 1% in the relative errors. In the verification, the absolute error is 3.2 mg/L of algae and a relative error of 17.86. The change in the relative error is insignificant. However, in the absolute error, the reduction is 1.75 mg/L of algae.

With the absolute and relative errors calculated, we then tested with the use of a neural network.

### 4.5. Use of Neural Network

**Figure 20:**
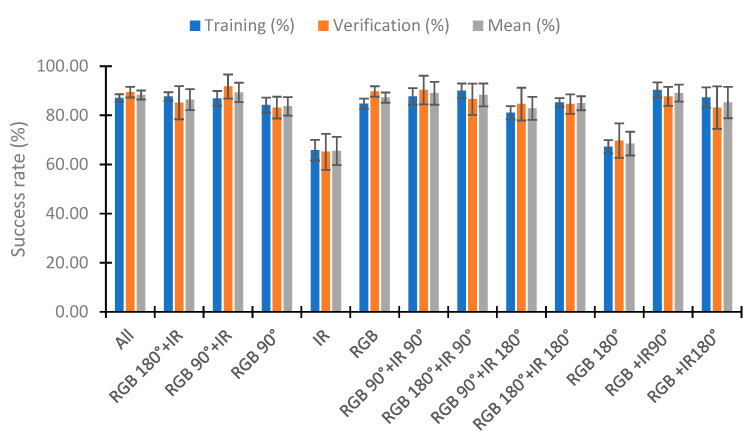
Success rate in the neural network with the use of IR and RGB lights in the different angles.

In this section, we analyze the use of a neural network to classify the percentage of algae in the samples with the neural network nearest neighbor. To perform the test, we used 110 random samples to train the neural network and the rest (70 samples) to verify. This process was repeated five times, and the training and verification means were calculated. Figure 20 represents the mean of the five repeats in relation to the combination of IR light and RGB light in the different angles tested. Figure 20 represents these combinations as the lights and angles used. If the category does not include an angle, it is the use of the two angles (e.g., RGB+IR90 is the use of the RGB light at angles 90° and 180° with the combination of the infrared light at 90°). The RGB 180° and IR categories present bad results, with a success rate of 67.27% and 65.82% in training, respectively. Success rates higher than 80% are observed in the other combinations in the training and verification. The better results are seen in the combination of all lights (all), RGB90° + IR, RGB + IR90°, RGB 180° + IR90°, and RGB 90° + IR90°, with hit rates higher than 88% in the mean of the training and verification. In training, the combinations RGB 180° + IR90° and RGB + IR90° have a success rate of 90%. However, they present a lower success rate in verifying, with success rates of 86.57 and 87.71%, respectively. The better results in the verification are in the combination RGB 90° + IR, with the success rate being 91.71%. Although the % for correctly classified in training is 86.91%, this configuration is the one that presents the best average between the calibration and the verification, with a value of 89.31%. Additionally, the calibration presents the slightest deviation, with 1.49%, and the second most minor deviation in the verification, with 2.17%.

**Figure 21:**
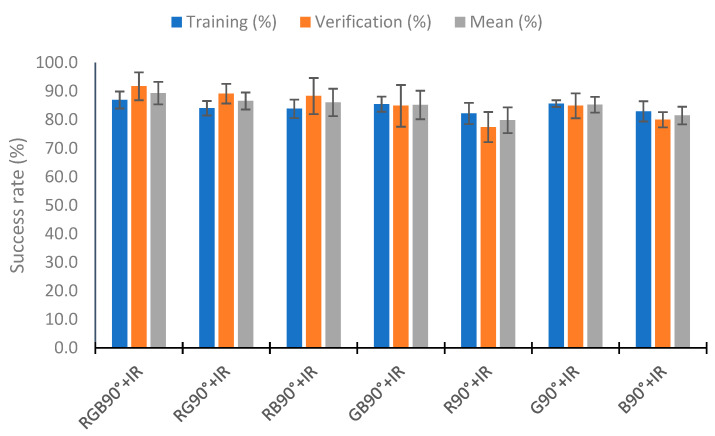
Success rate in the neural network with the data at 90° of the RGB light and IR light.

Now, we analyze the reduction in the data in the better configuration (except All). The first analyzed is RGB 90° + IR (Figure 21). We tested the configuration RGB 90° + IR with different combinations of visible light (using one or two visible wavelengths). The worst results are in the use of red or blue light with IR. In these cases, the success rate in calibration is less than 82.9%. The results are better for using two lights than for using only one light. However, the better results are in the combination of RG 90° + IR with a mean success rate of 86.57%. This is less than the 89.31% of success rate seen with the use of three lights (RGB 90° + IR).

**Figure 22:**
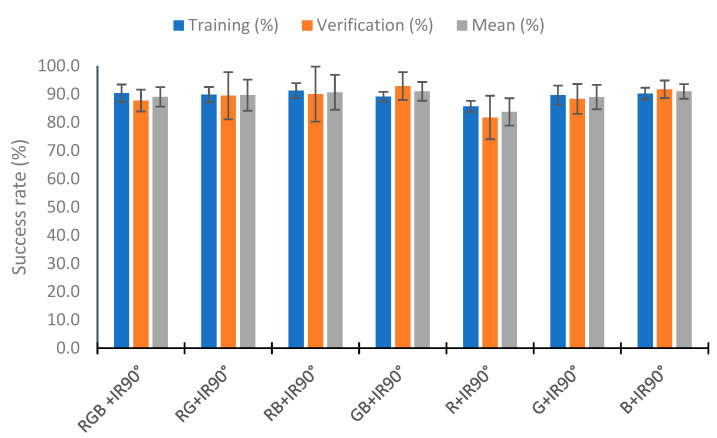
Success rate in the neural network with the data of the different RGB light and IR light at 90°.

Figure 22 represents the results obtained by eliminating visible wavelengths in the combination of the IR90° light. Except for the configuration of R + IR90°, with success rates of 85.6% in the verification and 81.7% in the verification (a mean of 83.7%), the results are similar. The maximum improvement in the mean is 2% in the configuration GB + IR90°. In the RB + IR90° combination, there is an improvement in the training and verification, and in the GB + IR90° and B + IR90° combinations, the results in the training are worse, but in the verification they are better. According to the standard deviation, the configuration RB + IR90° presents the maximum deviation in the verification with a value of 9.74%. In this configuration, the better results are in the configuration B + IR90°. Although it has a diminution of 0.2% in the success rate compared with the use of RGB + IR90°, the results in the verification are improved by 4% (success rate of 91.7% in the verification), and the mean of the verification and calibration is 90.9%. This value is similar to that obtained in the configuration GB + IR90° (91.0%). However, the configuration RGB + IR90° has a standard deviation of the results that is less.

**Figure 23:**
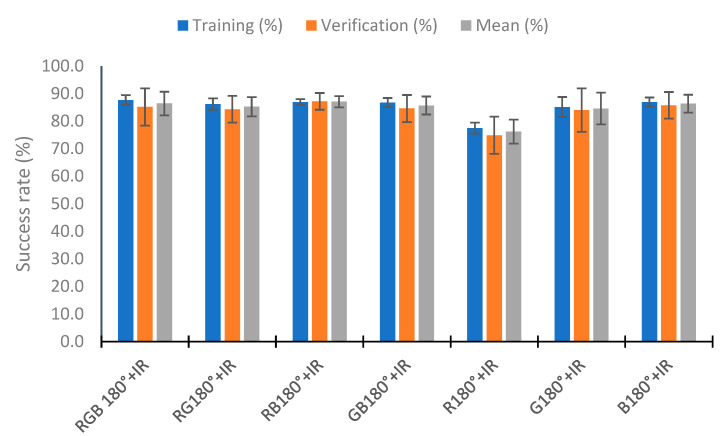
Success rate in the neural network with the data of the different RGB light at 180° and IR light.

Figure 23 shows the results obtained with the reduction in the data in the configuration RGB 180° + IR. As in the previous cases, the use of red light together with infrared is the one that provides the worst results. There is a deterioration in the classification by approximately 10% in the calibration and verification. In the other configuration, the results are similar, the minimum success rate in training is 85.1% (G180° + IR), and the maximum is 87.6% (RGB 180° + IR). The results in the verification are similar. However, in the standard deviation, the configuration RB 180° + IR has less deviation. There is a mean deviation of 2.03% for the success rate. In the other case, the values obtained are higher than 3.25%.

**Figure 24:**
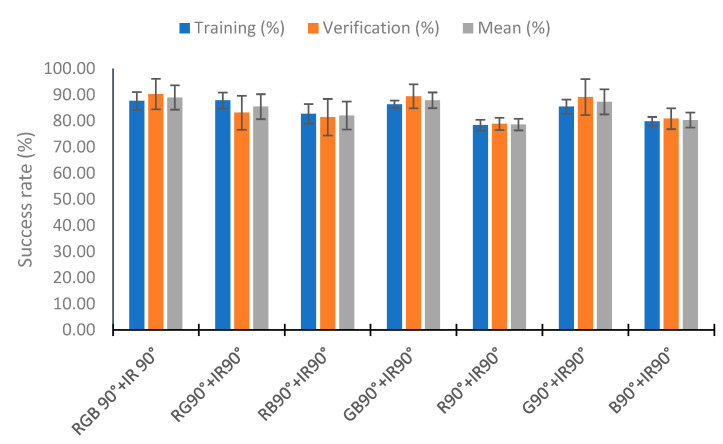
Success rate in the neural network with the data of the different RGB and IR light at 90°.

Finally, we analyze the configuration RGB 90° + IR90° in Figure 24. In this case, the worse results are in the configurations R90° + IR90° and B90° + IR90°. These configurations have success rates in the training of 78.4% in R90° + IR90° and 79.8% in B90° + IR90°. The better results in the training are in the configuration RG90° + IR90°, with a success rate of 87.8%, and the second in RGB 90° + IR90°, with a value of 87.64%. The better results in the verification and mean are in the configuration RGB 90° + IR90°, with a value of 90.29% and 88.96%, respectively. Finally, the lower standard deviation in the configuration with higher values for the mean success rate is in the configuration GB90° + IR90°, with a mean standard deviation of 3.01%. In this configuration, the mean success rate is 87.9%. As the results are similar for the success rate, but there are fewer deviations in the training and verification, the better configuration is GB90° + IR90°.

Once we tested with the different configurations, we reached the conclusion that the better configurations are B + IR90°, with a mean success rate of 90.9%, and RGB90° + IR, with a performance of 89.3%.

## 5. Conclusions

Eutrophication is a problem that can affect all water bodies. There are techniques to determine the presence of chlorophyll a. These techniques are costly or cannot be used in continuous monitoring. So, the use of turbidimeters is a solution to control the presence of algae in the water. However, traditional turbidimeters cannot differentiate between turbidity from sediments and turbidity from algae.

Our prototype uses IR and RGB light to monitor the turbidity of the water and the source of turbidity. Our prototype uses two photoreceptors located at 90° and 180° to detect scattered and absorbed light, respectively.

Using the IR light at 90°, our results show a good relationship (R^2^ = 0.983) between the sensor response and turbidity. We obtained a Vout difference of 0.658 V between the minimum and maximum turbidity tested. The errors to determine the turbidity with this light is 4.04 NTUs and a relative error of 8.90% in the calibration and 2.85 NTUs and a relative error of 7.45% in the verification for NTU readings higher than 2.73 NTUs. The IR light used at 180° responds more to the increase in the solid concentration (R^2^ = 0.994) than turbidity. In this case, the Vout difference between 0 to 200 mg/L is 1.796 V with an absolute error of 4.16 mg/L and a relative error of 8.09% in the calibration. In the verification, the absolute error is 3.79 mg/L and with a relative error of 11.40%.

Using RGB light, we observe differences between the different samples. For the same concentration, the Vout is higher in the concentration that has more % of algae. As a result of the information on the concentration and turbidity obtained with IR light, we obtain a calibration error of 2.78 mg/L of algae and a relative error of 13.91% in the calibration. In the verification, these errors are 3.20 mg/L of algae and 17.86%.

Finally, we tested with the use of a neural network. We obtained that using blue light at 90° and 180° in the combination of IR at 90° has a success rate of 90.9%.

In future work, we will test the prototype in real conditions that will allow for increasing the technology readiness level (TRL) and the use of other lights such as ultraviolet and an RGB combination to detect other alga species or substances such as organic matter. In the case of substances that can absorb light in the regions of RGB or UV, combining the different lights reduces this problem. This is because, usually, the substances absorb in determined wavelengths, and their absorbance is near to zero in other wavelengths. To test in real conditions, we are going to waterproof the system. In the future, this prototype can be used to control eutrophication in sensitive areas to minimize the negative effects of eutrophication, such as the loss of water attractiveness, environmental damage, and problems in water supply, among other issues.

## Figures and Tables

**Table 1:** Resistance and intensity in IR LED and the resistance in the voltage divider.

Resistance LED (Ω)	1200	560	220	150	100	68	33
Intensity LED (mA)	3.5	7.09	17.73	24.14	35.29	53	100
Resistance photodiode (kΩ)	100	330	1000	3000	8200	10,000	

**Table 2:** Selection of the better resistance in the LDR voltage divider.

Parameters	Red 180°	Red 90°	Green 180°	Green 90°	Blue 180°	Blue 90°
Resistance 0 mg/L (kΩ)	1.04	16.25	0.72	16.61	1.09	27.12
Resistance 100 mg/L (kΩ)	1.24	84.70	0.97	40.71	1.53	65.70
Optimal resistance (kΩ)	1.14	37.10	0.84	26.00	1.29	42.21
Voltage difference (V)	0.153	1.280	0.245	0.707	0.280	0.685
Select resistance (kΩ)	1.20	33.00	1.20	33.00	1.20	33.00
Voltage difference (V)	0.145	1.276	0.238	0.698	0.280	0.674

**Table 3:** Error of mathematical models of the IR light at 180° of the photodiode.

	Equation (3)		Equation (4)	
	Absolute (mg/L)	Relative (%)	Absolute (mg/L)	Relative (%)
Error calibration	5.69	11.14	4.16	8.09
Error verification	3.90	11.84	3.79	11.40

**Table 4:** Errors of models with IR and RGB at 90°.

	Equation (5)		Equation (6)	
	Absolute (mg/L)	Relative (%)	Absolute (mg/L)	Relative (%)
Error calibration	5.73	19.17	6.85	29.49
Error verification	6.78	30.58	8.62	53.33

**Table 5:** Absolute and relative errors in the calibration and verification of the models G and H.

	Equation G		Equation H	
	Absolute (mg/L)	Relative (%)	Absolute (mg/L)	Relative (%)
Error calibration	3.74	14.98	3.99	11.61
Error verification	4.95	17.95	5.63	25.53

**Table 6:** Absolute and relative errors in the calibration and verification of model 7 improved.

	Absolute (mg/L)	Relative (%)
Error calibration	2.78	13.91
Error verification	3.20	17.86

## Data Availability

The data presented in this study are available on request from the corresponding author. The data are not publicly available due to privacy constraints.
